# Cryptogenic Acute Fibrinous and Organizing Pneumonia: A Case Report

**DOI:** 10.7759/cureus.43563

**Published:** 2023-08-16

**Authors:** Dorcas Lim, Sok Boon Tay

**Affiliations:** 1 Department of Internal Medicine, Sengkang General Hospital, Singapore, SGP; 2 Department of Respiratory Medicine, Sengkang General Hospital, Singapore, SGP

**Keywords:** subpleural nodule, pneumonia, pneumonitis, interstitial pneumonia, acute fibrinous organizing pneumonia

## Abstract

We describe the case of a middle-aged male who presented with incidental findings of a left retrocardiac opacity seen on a chest radiograph. Subsequent computed tomography (CT) of the thorax showed multiple subpleural lesions with central necrosis. A diagnosis of acute fibrinous organizing pneumonia was made histologically upon biopsy of the subpleural lesions. The patient responded well to steroids, with full resolution of the subpleural lesions on repeat CT of the thorax. This report illustrates a case of acute fibrinous organizing pneumonia wherein the patient responded well to corticosteroids.

## Introduction

Acute fibrinous organizing pneumonia (AFOP) is a rare entity of acute or subacute lung injury first described by Beasly et al. in 2002 [1]. AFOP is characterized by the presence of intra-alveolar fibrin deposition and organizing pneumonia without hyaline membranes. Diagnosis is often made via histology; however, optimal treatment for this disease remains unknown. Here, we describe a case of AFOP, the clinical picture, radiological and histological findings, and its treatment progress with corticosteroids. We feel this case is worth reporting as it contributes to the growing body of evidence for treating this disease.

## Case presentation

A 55-year-old male with no significant medical history presented to the Emergency Department with a two-day history of right subcostal pain. He was otherwise well with no respiratory, gastrointestinal, autoimmune, or systemic symptoms. He denied any recent travel or prolonged immobility. He had a 10-pack-year smoking history. There was no history of alcohol use, substance abuse, or chemical exposure. He worked as a bus driver.

**Figure 1:**
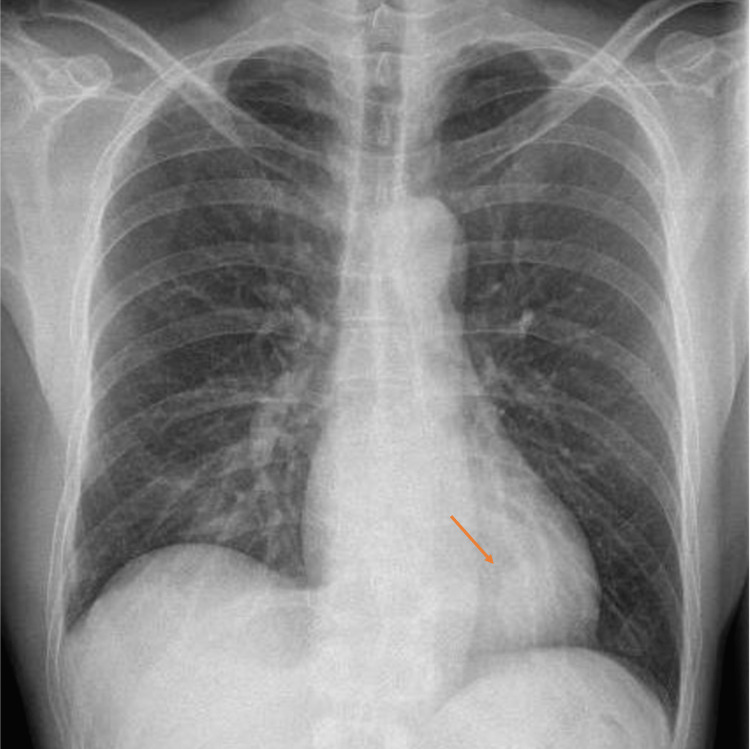
Chest radiograph on admission.

The patient was afebrile, both before and on admission. His heart rate was 68 beats per minute, blood pressure was 120/68 mmHg, and respiratory rate was 16 breaths per minute, with an oxygen saturation of 96% on room air. Clinical examination revealed dual heart sounds with no murmurs and good air entry over bilateral lungs. Abdominal examination was unremarkable. No calf edema or tenderness was noted. Chest X-ray (CXR) showed a 2.7 cm left retrocardiac density, as seen in Figure 1.

Initial laboratory data are presented in Table 1.

**Table 1:** Initial laboratory investigations

Parameter	Results	Normal range
Hemoglobin (g/L)	12.4	14–18
White blood cell count (L)	10.79 × 10^9^	4–10 × 10^9^
Neutrophils (%)	71.1	40–75
Lymphocytes (%)	23.6	15–41
Eosinophils (%)	1.1	0–6
C-reactive protein (mg/L)	158	<5
Erythrocyte sedimentation rate (mm/hour)	61	1–10
HbA1c (%)	5.8	<6.5
HIV screen	negative	-

Subsequently, he developed low-grade fevers of up to 37.8° daily. In view of the low-grade fevers during admission and mildly raised inflammatory markers, oral antibiotics were initiated.

**Figure 2:**
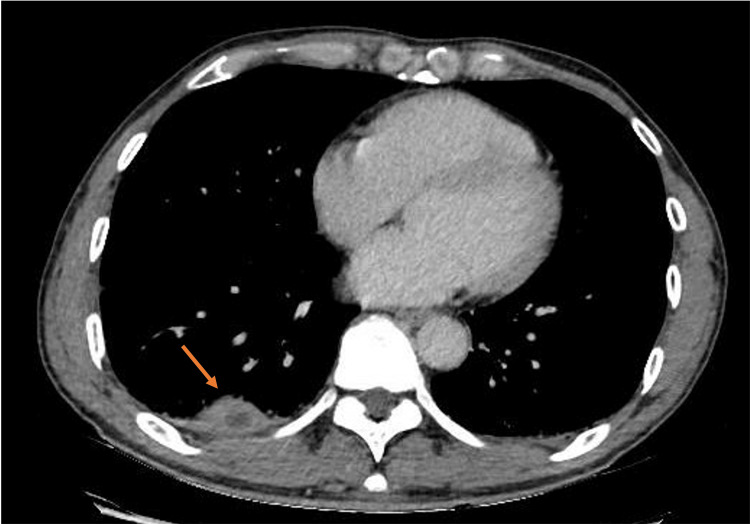
Computed tomography of the thorax showing a subpleural lesion with central necrosis in the right lower lobe.

**Figure 3:**
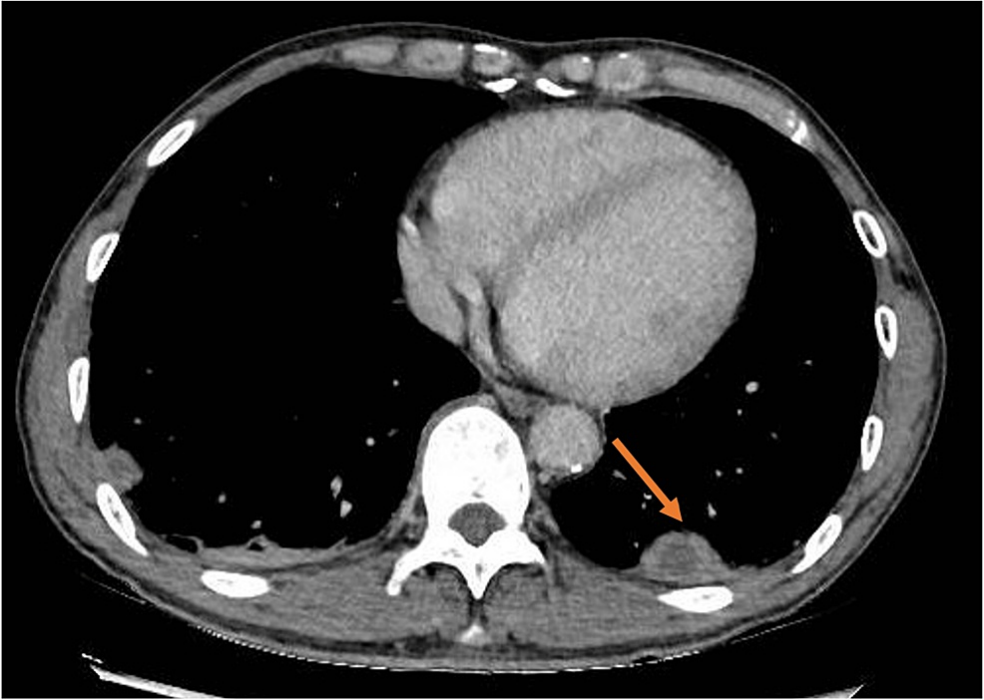
Computed tomography of the thorax showing a subpleural lesion with central necrosis in the left lower lobe.

A computed tomography (CT) of the thorax was performed in view of his CXR findings which showed multiple subpleural lesions in bilateral lower lobes, some of which had small central necrotic areas. No enlarged mediastinal, hilar, axillary, or supraclavicular lymphadenopathy was detected, as shown in Figure 2 and Figure 3.

He underwent a CT-guided biopsy of the thoracic lesions. Histology revealed the presence of recurrent alveolar hemorrhage, alveolar and capillary fibrin deposits, and mild-to-moderate focal interstitial and perivascular lymphocytic infiltrate, supportive of acute fibrinous organizing pneumonitis.

Autoimmune screening including anti-neutrophil antibody, anti-extractable nuclear antigen antibody, anti-neutrophil cytoplasmic antibody, rheumatoid factor, and anti-cyclic citrullinated peptide antibody was negative. Sputum studies were negative for microbiology and acid-fast bacilli.

The patient continued to spike low-grade fevers despite completing a course of oral antibiotics. He was offered a bronchoscopy with bronchoalveolar lavage to rule out possible concomitant infections. However, the patient declined the procedure. Given the overall clinical findings and histology results, he was started on high-dose oral prednisolone of 1 mg/kg for one month, followed by a taper of 10 mg every fortnight. The low-grade fever subsequently lysed within four days of corticosteroid initiation.

**Figure 4:**
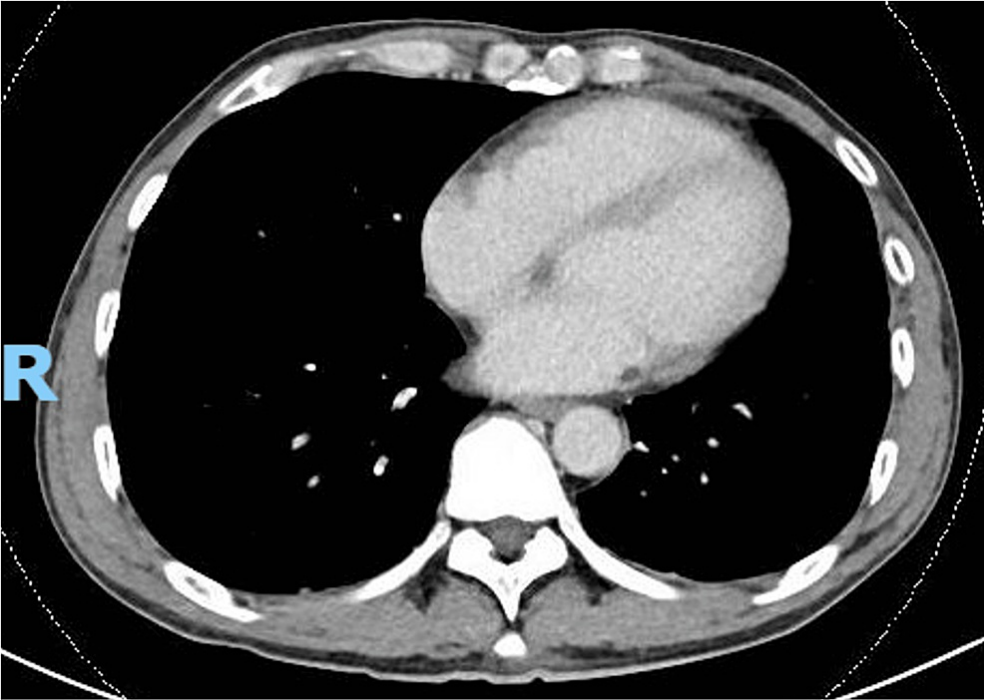
Computed tomography of the thorax showing resolution of the subpleural lesion in the right lower lobe.

**Figure 5:**
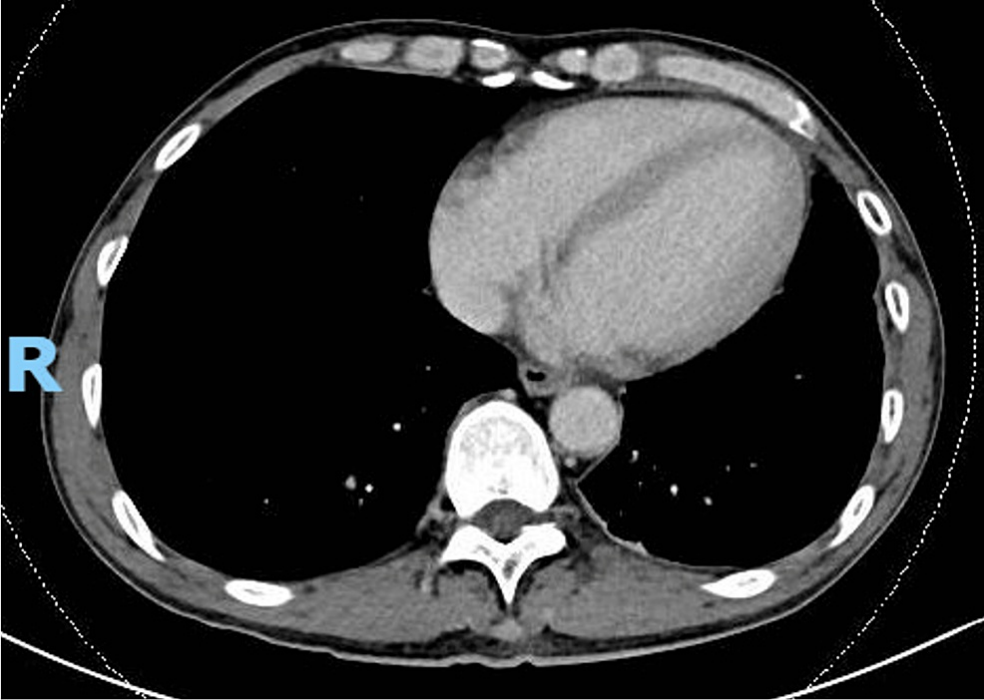
Computed tomography of the thorax showing resolution of the subpleural lesion in the left lower lobe.

Follow-up CT imaging revealed full resolution of the subpleural lesions at two months of treatment, as shown in Figure 4 and Figure 5.

**Figure 6:**
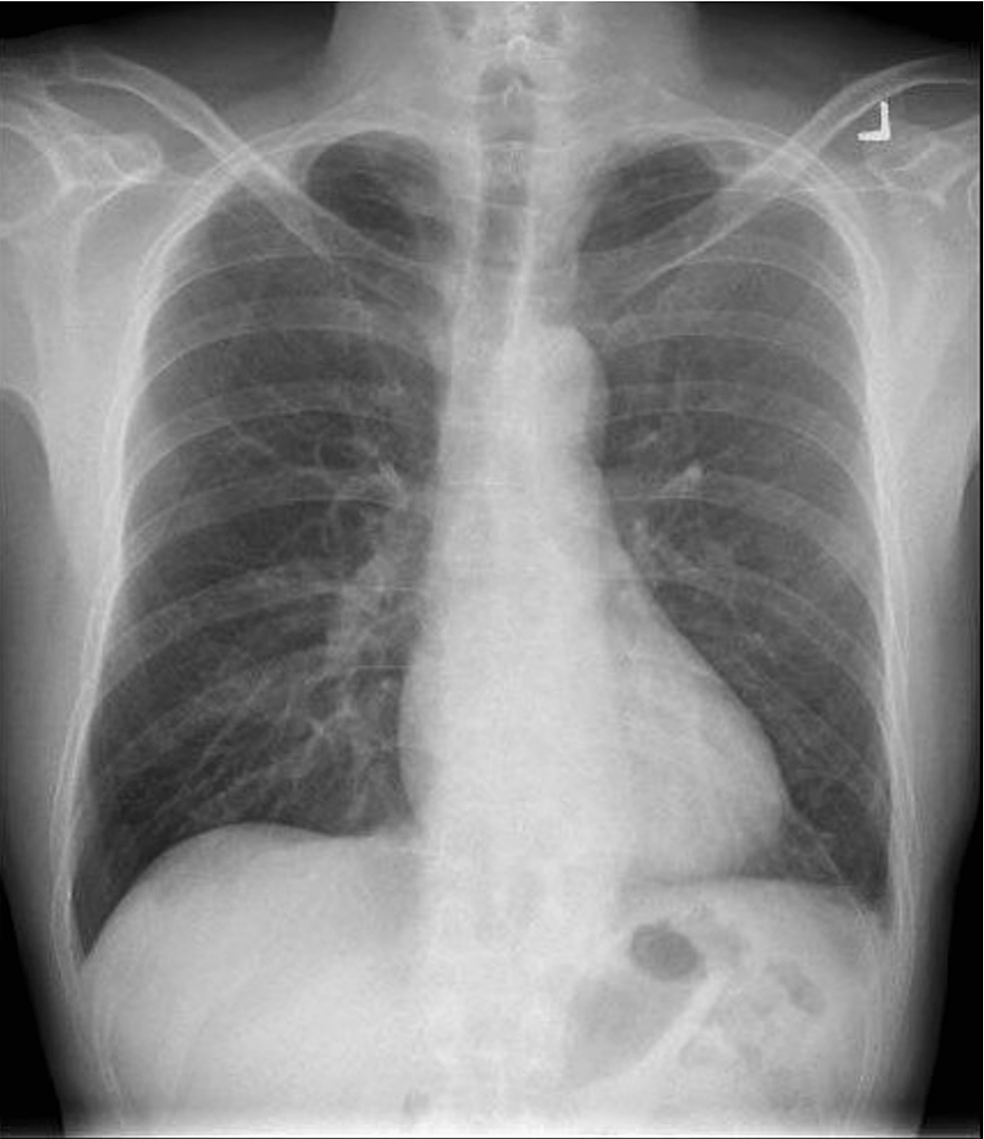
Chest X-ray showing resolution of the left retrocardiac density.

**Figure 7:**
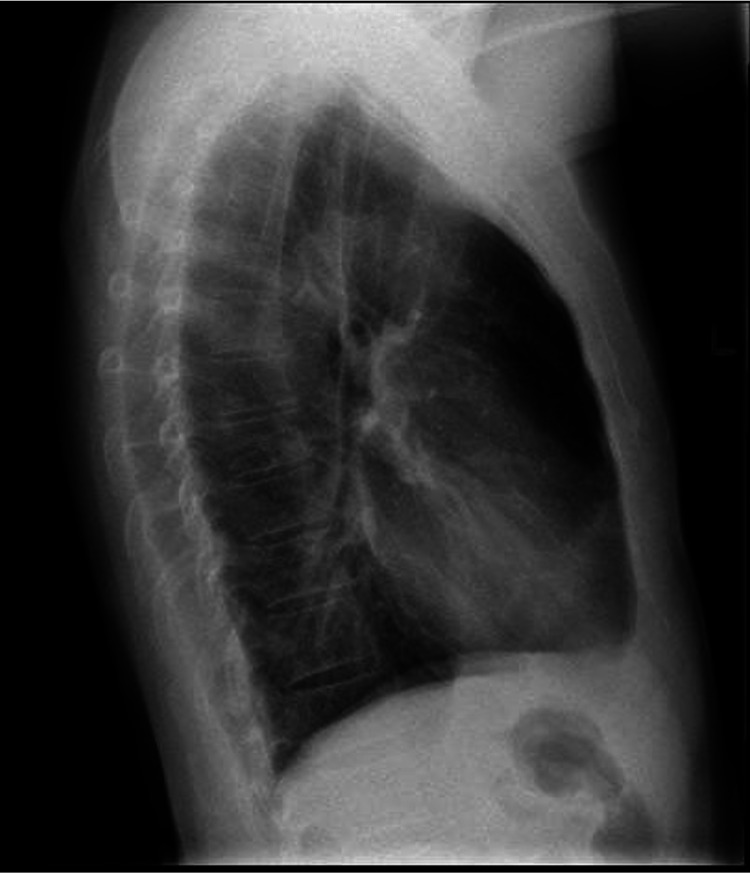
Lateral chest X-ray showing resolution of the left retrocardiac density.

A repeat CXR at four months showed no recurrence of the lesion, as shown in Figure 6 and Figure 7.

## Discussion

AFOP is a rare and distinct histopathological pattern of lung injury that falls under the broader category of idiopathic interstitial pneumonia. It was first described by Beasly et al. in 2002 [1]. It is characterized by the presence of intra-alveolar fibrin deposition and organizing pneumonia [2].

The exact cause of AFOP is not always clear. Overall, 56% of patients with AFOP recruited in a study had no underlying etiologies identified [3]. However, various associations were observed, including autoimmune diseases, infections, drugs, and occupational exposures, as listed in Table 2 [1,2,4].

**Table 2:** Associations seen with acute fibrinous organizing pneumonia.

Classification	Examples
Autoimmune diseases	Ankylosing spondylitis, anti-phospholipid syndrome, anti-synthetase syndrome, dermatomyositis, Sjogren’s syndrome, systemic lupus erythematosus, polymyositis, primary biliary cirrhosis
Infections	Acinetobacter baumannii, Aspergillus fumigatus, Chlamydia pneumoniae, cytomegalovirus, H1N1influenza, Haemophilus influenzae, histoplasmosis, human immunodeficiency virus, Pneumocystis jirovecii
Drugs	Abacavir, amiodarone, bleomycin, decitabine, everolimus, sirolimus, Az acytidine
Environmental	Aerosols, asbestos, coals, dust
Transplant	Allogenic hematopoietic stem cell transplant, lung transplant

AFOP may present with fever, cough, dyspnea, and respiratory failure with acute respiratory distress syndrome, while others may remain asymptomatic [5]. Most patients receive a course of antibiotics to treat the underlying lung infection. However, in AFOP, they are unlikely to make significant improvements with antimicrobial therapy alone, as seen in our patient, who continued to have low-grade fevers despite antibiotics. Hence, failure of clinical response to antibiotics for the treatment of pneumonia may also be a presentation of AFOP [6].

Our patient presented with an isolated left retrocardiac opacity on CXR. The initial differentials considered included infections such as pneumonia and tuberculosis, malignancy, and benign neoplasms such as hamartomas.

Radiologically, while the findings of bilateral basal opacities with areas of consolidation predominate on CT, there are no specific radiological features that are pathognomonic for AFOP [2]. Bilateral diffuse consolidation with ground-glass changes and multifocal nodules were also seen on CT in patients with histologically proven AFOP [7].

Histological diagnosis is crucial for the diagnosis of AFOP, as evidenced by our case, where steroids were initiated upon the availability of histology results. Histology is also valuable for helping to rule out differentials such as tuberculosis, vasculitis, and malignancy. Lung biopsy is preferred and can be obtained via CT-guided lung biopsy or video-assisted thoracoscopic lung biopsy. Bronchoscopy with bronchoalveolar lavage often yields non-specific findings and may not provide a definitive diagnosis [8]. However, it may help rule out any underlying infections before initiating high-dose corticosteroids or immunosuppression.

Histologically, intra-alveolar fibrin deposition is the main finding seen in AFOP. This pattern may also be seen in diffuse alveolar disease (DAD), organizing pneumonia (OP), and eosinophilic pneumonia (EP). Therefore, the presence of a hyaline membrane in DAD, intra-alveolar fibroblastic plugs (Masson bodies) in OP, and eosinophils within the tissue in EP help differentiate between the various diagnoses [7].

Since AFOP was first reported in the literature in 2002, several cases have been reported. However, there has yet to be a standardized guideline for managing AFOP [8]. Management of AFOP involves a multimodal approach, including supportive treatment with oxygen therapy as needed, corticosteroids, and immunosuppressants if there are associated autoimmune diseases or immune-related conditions [4]. Corticosteroids remain the mainstay of treatment for AFOP, with positive outcomes reported in multiple case reports [7,9-11]. As there is currently no consensus on dose and treatment duration, these should be guided by the therapeutic response, both clinically and radiologically.

## Conclusions

AFOP is a rare entity of interstitial pneumonia. The differential of AFOP should be considered in patients who failed appropriate antimicrobial treatment for underlying lung infections. Radiographic features are non-specific and can mimic other more common diagnoses, such as infection, neoplasm, or other interstitial lung diseases. Therefore, the diagnosis can be missed easily if the patient was treated for other conditions and remains asymptomatic. Failure to improve clinically or radiologically should raise the index of suspicion, with consideration for biopsy as the histological examination is paramount for the diagnosis of AFOP. Steroid therapy remains the mainstay of therapy, although optimal doses and duration remain unknown. Frequent follow-up after initiation of treatment is prudent to assess clinical response, aiming to taper off steroids as soon as possible. Further immunosuppression may need to be considered for refractory cases, although in this case, the patient had complete resolution with corticosteroids.
